# How can malaria rapid diagnostic tests achieve their potential? A qualitative study of a trial at health facilities in Ghana

**DOI:** 10.1186/1475-2875-9-95

**Published:** 2010-04-14

**Authors:** Clare IR Chandler, Christopher JM Whitty, Evelyn K Ansah

**Affiliations:** 1 Clinical Research Unit, London School of Hygiene & Tropical Medicine Keppel Street, London, WC1E 7HT, UK; 2 Dangme West District Health Directorate, Ghana District Health Service, PO Box DD1, Dodowa, Ghana

## Abstract

**Background:**

Rapid diagnostic tests (RDTs) for malaria are at the early stages of introduction across malaria endemic countries. This is central to efforts to decrease malaria overdiagnosis and the consequent overuse of valuable anti-malarials and underdiagnosis of alternative causes of fever. Evidence of the effect of introducing RDTs on the overprescription of anti-malarials is mixed. A recent trial in rural health facilities in Ghana reduced overprescription of anti-malarials, but found that 45.5% patients who tested negative with RDTs were still prescribed an anti-malarial.

**Methods:**

A qualitative study of this trial was conducted, using in-depth interviews with a purposive sample of health workers involved in the trial, ranging from those who continued to prescribe anti-malarials to most patients with negative RDT results to those who largely restricted anti-malarials to patients with positive RDT results. Interviews explored the experiences of using RDTs and their results amongst trial participants.

**Results:**

Meanings of RDTs were constructed by health workers through participation with the tests themselves as well as through interactions with colleagues, patients and the research team. These different modes of participation with the tests and their results led to a change in practice for some health workers, and reinforced existing practice for others. Many of the characteristics of RDTs were found to be inherently conducive to change, but the limited support from purveyors, lack of system antecedents for change and limited system readiness for change were apparent in the analysis.

**Conclusions:**

When introduced with a limited supporting package, RDTs were variously interpreted and used, reflecting how health workers had learnt how to use RDT results through participation. To build confidence of health workers in the face of negative RDT results, a supporting package should include local preparation for the innovation; unambiguous guidelines; training in alternative causes of disease; regular support for health workers to meet as communities of practice; interventions that address negotiation of health worker-patient relationships and encourage self-reflection of practice; feedback systems for results of quality control of RDTs; feedback systems of the results of their practice with RDTs; and RDT augmentation such as a technical and/or clinical troubleshooting resource.

## Background

Rapid diagnostic tests for malaria are being put forward as a potential solution for targeting valuable anti-malarial drugs to those who need them [1]. Malaria overdiagnosis, and misdiagnosis, is now recognized across malaria endemic countries in Africa and Asia [2]. The consequences of overdiagnosis include poor health outcomes due to missed diagnoses of alternative causes of symptoms [3] and exposure to unnecessary medication, wastage of valuable drugs and unnecessary expenditure at the household, country health system and donor levels [4,5]. The consequences of missed cases of malaria are avoidable morbidity and mortality, and it is important than measures to reduce overdiagnosis do not lead to an increase in the number of missed malaria cases. Malaria overdiagnosis has been reported at primary health care providers [6,7] as well as at hospitals [3,8,9]. Whilst improved microscopy can in principle provide better diagnostic support in well resourced settings such as hospitals, there is a strong argument for the introduction of simple, fast and relatively cheap rapid diagnostic tests (RDTs) in low resource settings [1]. Many malaria endemic countries are now undertaking the introduction of RDTs at various levels of the public and private health sectors.

Conclusive evidence about how RDTs could achieve improved health and economic outcomes in operational practice is lacking, but initial evidence suggests simply deploying them with a minimal training package will have an impact well below their potential and may have no impact at all. Important assumptions underlying the potential cost-effectiveness of their introduction are that the tests will be used on all febrile patients and that test results will be adhered to; where this is not the case their cost-effectiveness drops rapidly [10]. There are mixed results from efforts to introduce RDTs. Where they have been introduced with a relatively intensive programme, including training in guidelines with recommendations for alternative treatment for RDT negative patients, regular supportive supervision, and even incentives for participation in the study, successes have been reported [11-15]. However, where RDTs have been introduced with support more typical of the training package a Ministry of Health can roll-out rapidly, between 35% and 55% RDT negative patients have been prescribed anti-malarials [16-19]. In one study in Burkina Faso, as many as 85% RDT negative patients were prescribed anti-malarials in spite of a strong message that a negative RDT virtually excludes clinical malaria [20]. Thus, whilst training messages are likely to play a role in adherence to RDT results, it is clear that factors beyond training are also important. More detailed process evaluations of RDT interventions are required to identify factors most important for the successful introduction of RDTs. In Africa the question is, therefore, moving from whether RDTs have an impact, to reasons why they are effective in some settings and not others, with the aim of maximising their impact.

This study aimed to address this gap with a qualitative study of the introduction rapid diagnostic tests to public health facilities in a rural district of Ghana. The quantitative results of the trial [16] found that where microscopy was present, the addition of RDTs had no impact on the prescription of antimalarial drugs according to test results. Where current diagnosis was clinical (without tests) the introduction of RDTs led to a significant reduction of over-prescription of anti-malarials, but anti-malarials were still prescribed to half of all patients who had negative RDT results. Across the microscopy and clinical health facilities, 45.5% RDT negative patients received anti-malarials, with some variation between health facilities and between health workers. RDTs, therefore, had an impact, but well below their potential maximum. The objective of the qualitative study reported in this paper was to understand how RDTs were integrated into practice through an understanding of the meanings attributed to RDTs by different health workers.

## Methods

### Study setting

The trial took place at four health facilities in the Dangme West District of southern Ghana, a purely rural district with an estimated 2008 mid-year population of 136,622. The population lives in scattered small communities of less than 2,000 people and there is widespread poverty in the district. The main local language is *Dangme*. The district is divided into four administrative sub-districts. There are a total of 17 health facilities serving the population. These include four health centres and six community clinics in the public sector. Private sector facilities include three private clinics and two private maternity homes. There is also a mission clinic as well as a quasi-government clinic. The doctor:population ratio is 1: 19,517. There are three publicly owned laboratories and two privately owned laboratories. One of the health centres was in the process of being upgraded to a district hospital status at the time of the study. Malaria accounts for about 50% of all reported cases at the outpatients department in all health facilities and most of these cases are as a result of presumptive diagnosis. In the three health facilities where laboratory facilities did exist, on average, 68% of malaria diagnosis was confirmed by a laboratory test at the time of the study. The health facilities in the study varied in size and composition: health facility III (HFIII) was a large health centre with a high patient load, one medical doctor and 4 medical assistants providing prescriptions as well as 10 nurses. Health facilities facility I and II (HFI and HFII) were smaller. One was a small health centre (HFII) whilst the other was a community clinic (HFI). Both had five prescribers, including one medical assistant at the health centre. Health facility IV (HFIV) was a private clinic with one medical assistant prescribing.

The trial which preceded this study randomly allocated febrile patients at the four health facilities to receive either an RDT or current diagnostic (microscopy in one health centre and clinical judgement in the three other facilities). The RDT was carried out by a member of the research team stationed at the health facility in a separate room from the consultation or the laboratory where there was one. Patients were therefore identified as needing a test by the health worker and sent to the research team for enrolment in the study and randomization to RDT or existing diagnostic method. The patient was then sent back to the health worker for review together with a written result for the RDT and the RDT cassette itself or routine result form in the case of microscopy.

RDTs were introduced to health workers at participating health centres at an initial workshop which focused on how to do an RDT as well as revision of clinical signs and symptoms of malaria and other common diseases presenting with fever among adults and children. Health workers who were transferred to any of the health facilities during the trial were given a one-to-one introduction to RDTs using the same training package. The trial intended to assess whether the provision of RDTs was sufficient to change practice and so the research team deliberately avoided giving advice on clinical practice in relation to RDT results during the trial. If asked, the research team was instructed to advise health workers to treat patients according to their own judgement of the correct practice.

### Trial

The qualitative study was conducted one month after the trial of RDTs had ended (February 2009) and consisted of in-depth interviews with health workers who had participated in the trial, in-depth interviews with stakeholders from the district health management team and focus group discussions with patients who had been enrolled in the trial. Findings from the patient focus group discussions will be reported separately.

### Sampling

All health workers at participating health facilities were eligible for interview as long as they had enrolled patients into the trial. A total of 29 health workers participated in the trial, including 16 at the largest health centre of whom several had used RDTs infrequently. Participation in the qualitative study was based on frequency of prescribing. The four health workers prescribing most frequently at the large health centre were selected, the three most frequent prescribers were selected at the two smaller health facilities and the only prescriber at the private clinic was selected, making a total of 11 interviewees selected. If the selected health worker was unavailable during the period of research, the next most frequent prescriber was invited. Two stakeholders were identified for interview as members of the district health management team with a special interest in malaria. Preliminary analysis of transcripts was conducted daily to assess saturation of data and allow for expansion of the sample size.

### Conducting interviews

Each potential participant was contacted by the study team by phone or face-to-face visit to invite them to the interview. All interviews were conducted by CC, who was introduced as a non-clinician researcher and was not involved in the original trial in a private space outside of the health facility after giving information about the study and gaining consent from the participant. Interviews were conducted in English and were tape-recorded. Interviews followed a topic guide that focused on the use of RDTs. For the health workers, the following domains were covered: logistics; trust; clinical signs compared to tests; peers; patients; use of anti-malarials; alternative causes of fever; restricting anti-malarials and training. Domains for stakeholder interviews were: perception of RDTs; guidelines; campaigns; training; supervision; medical culture; following test results; RDTs and the future. Contact summary forms were completed after each interview to assist in assessment of topic saturation and to make any changes to the topic guide.

### Data analysis

Transcripts were made on the day of the interview or shortly after. Following principles of grounded theory [21], CC conducted line-by-line coding of ideas occurring in participant responses using the NVivo qualitative data analysis package (QSR International). Idea codes, such as 'I tried restricting anti-malarials and followed-up patients myself' were grouped into themes, such as 'experimentation'. Theories of behaviour change were reviewed and the themes in the data were grouped into theoretical concepts, such as 'learning through participation', based on the data together with wider bodies of theory.

### Ethics

The ethical review boards of Ghana Health Service and the London School of Hygiene & Tropical Medicine approved the study. The trial which preceded this study was prospectively registered at ClinicalTrials.gov NCT00493922.

## Results

There were no refusals to participate; three health workers were away from the district and could not participate and in those cases the next most frequent prescribers were invited to participate. Quotes from participants are cited with pseudonyms.

### The innovation in communities of practice

Analysis of themes emerging from the interviews suggested that a process of interaction with tests and with other actors underlay health worker conceptualizations of RDTs and their use. Wenger's theory of social learning [22] was found to be particularly relevant in the way respondents had engaged with RDTs in this study. Wenger focuses on learning as social participation, with groups engaged in the pursuit of shared enterprises learning through '*communities of practice*'. Mechanisms of learning take place through participation and reification. In *participation*, an individual engages in action (a process of taking part) and connection (relations with others that reflect the action). Participation may involve others who are not present, colleagues (such as co-prescribers) who are 'looking over your shoulder, as it were, representing for you your sense of accountability to the professional standards of your community'. In *reification*, an abstraction that has been made in order to make sense of experiences is treated as though it exists as a concrete entity by a group of individuals. Examples include laws or guidelines. The discourses of groups may support existing and emerging reifications. Discourse analysis is therefore important in understanding the reification of concepts that become accepted and shape meaning and participation. In this paper, learning in relation to malaria rapid diagnostic tests is deconstructed within these two theoretical constructs of participation and reification.

### Participation

**Figure 1:**
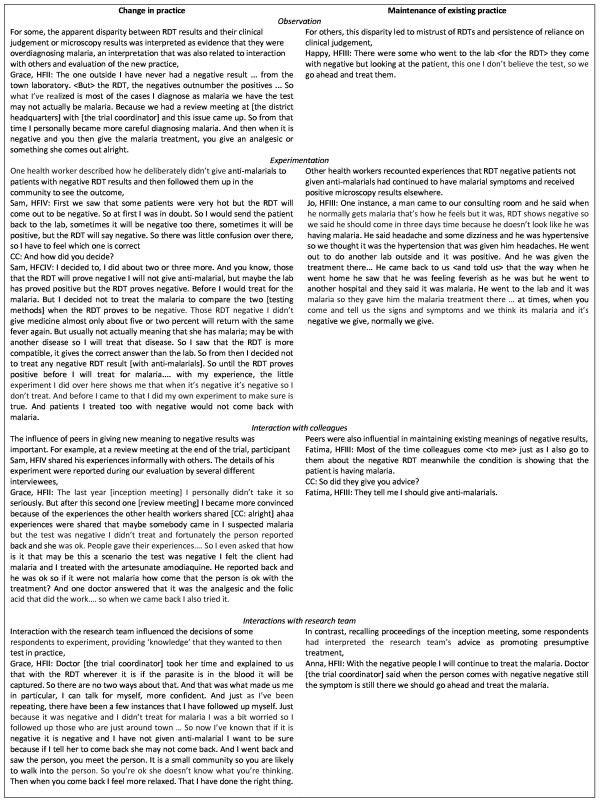
**Participation with RDTs in practice: as individuals and as groups**.

**Figure 2:**
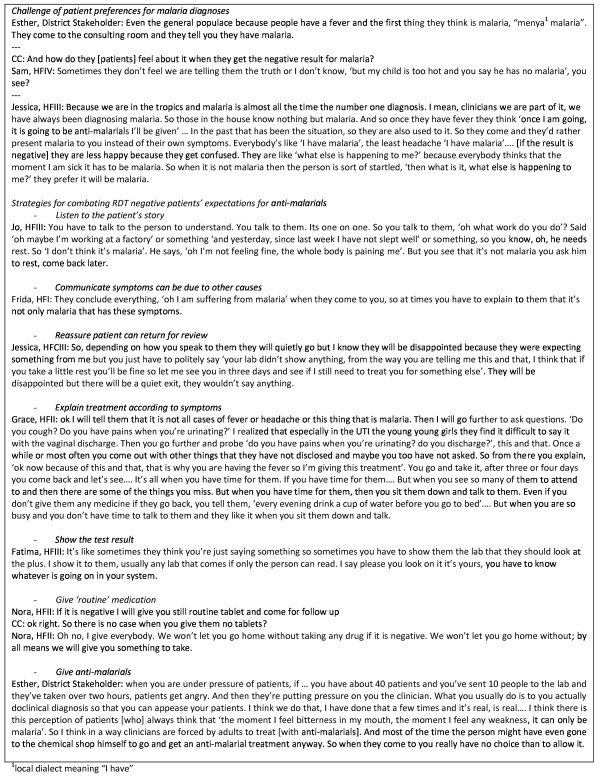
**Participation with RDTs in practice: with patients**.

Through participation and engagement with RDTs, health workers described a continuous process of negotiation of meaning of RDTs in their work and communities of practice. Figure 1 shows cases of health workers for whom observation, experimentation, interaction with colleagues and interaction with the research team were important in either changing or maintaining existing practice. Figure 2 shows the challenges of patient expectations for malaria diagnoses and strategies interviewees employed to tackle these challenges in the light of RDT results.

#### Interaction with RDTs

Learning occurred through participation with the RDTs and with others in health workers' communities of practice. All interviewees described how they had observed a difference between RDT results and their clinical judgement or microscopy results. For many, this led to mistrust of RDTs and a return to reliance on clinical judgement. Others, however, made more critical observations, such as a lack of improvement in patients prescribed anti-malarials with negative RDT results and improvement in patients who hadn't received anti-malarials with negative results. Following these observations, some health workers reported having experimented with changing their practice for patients with negative results. For some this convinced them to restrict anti-malarials to RDT positive patients. For others, however, experimentation led to uncertainty over validity of RDT results when patients, and even the respondents themselves, experienced persisting malaria symptoms after a negative result and had subsequently improved with anti-malarials.

#### Interaction with colleagues and researchers

In addition to their own observations and experimentations, health workers discussed how discussions with other health workers about their experiences and solutions to problems in practice had influenced their own conceptualization and practice with RDTs. For some, this led to greater confidence in RDT results; for others, communication with peers led to reassurance of confidence in clinical judgement over RDT results. Interactions with members of the research team also influenced health workers' understanding and use of RDTs, although understanding of the research team's statements varied amongst respondents.

#### Interaction with patients

In engaging with patients, and attempting to achieve successful consultations, further meaning of the RDTs was negotiated. Heath workers described how simply having and using RDTs improved patients' perceptions of the health facility and increased their willingness to attend the health centre,

'*The RDT facility that we are having here is making them to think that they have a new thing here ... So some of them will say 'when I went to [Health Facility II] Health Centre I went to lab'. They have been calling the RDT 'lab'. 'So they tested my blood and they said it is so so and so', so they are happy with it. Because when they fall sick and they come and we ask them to come there they're happy with it, it's ok*.' (Anna, HFII)

However, the presence of the tests alone was insufficient to lead to satisfactory consultations. Interviewees recognized that in negotiating the use of the new test, good relationships with patients was of great importance. Health workers described how discomfort associated with the RDT itself, such as waiting for the test result, could be reversed by the health worker's interpersonal skills,

CC: '*How do you think [waiting for the RDT result] will fit in with your practice?'*

Grace, HFII: '*It will depend... because you see a client, you've done the test, you asked him to wait for 15 minutes before you realized he's murmuring 'I have been here for a long time I've been sitting down'. But it all depends on the relationship. And then if you are able to explain very well. If you talk to them well and at times most of the clients have, the health workers, some have this language barrier, you can't communicate, communicates with them in the language they understand. And when you are using an interpreter it makes it more difficult translating from this one to this. But when they come and you speak the same language with them they feel more confident and it helps.'*

The greatest challenge in achieving successful consultations came when prescribing for patients with negative RDT results (Figure 2). Community members were perceived as holding onto the idea that all fever is malaria and sometimes to mistrust health workers who contradicted patients' assertions of malaria, preferring a diagnosis of malaria. Health workers described a number of strategies they developed to combat these expectations, including communicating (listening and explaining) well with the patient about their condition, test results or the treatment; reassuring the patient they can come back for review in subsequent days; explaining the treatment according to the patient's symptoms; showing the patient the test with the result and giving 'routine treatment', ensuring that patients would not be left without treatment to take home. In some cases, respondents reported that they felt no choice but to follow what they perceived the patient to want, and to prescribe anti-malarials.

### Reification

**Figure 3:**
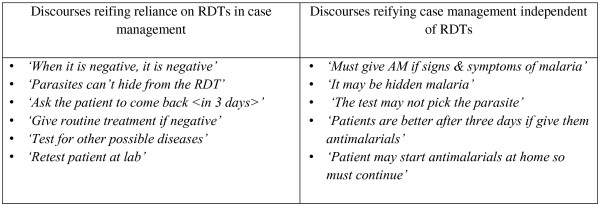
**Reification of meanings of RDTs**.

Participation with RDTs by colleagues within health workers' communities of practice led to the development of common meanings of the tests and results. These common meanings were reflected in discourses amongst health workers participating in the RDT trial. Discourses related to RDTs were found to 'reify', that is to concretise, ideas of either reliance on the tests or the non-significance of RDTs in the case management process (Figure 3). We observed that those who cited discourses reflecting reliance on RDTs also expressed pride with their new practice and, although the sample size was too small for statistical significance, quantitative analysis showed that these individuals also prescribed fewer anti-malarials to RDT negative patients compared to respondents who expressed mistrust with RDTs.

### Characteristics and context of the innovation

In constructing the different contextual factors, present and absent, in the introduction of RDTs to the trial population, a revised diffusion of innovations model by Greenhalgh *et al *[23] was found to be particularly useful. The model is derived from Rogers's [24] original framework based on a review of empirical evidence together with a review of behaviour change theories in service organizations. Different levels of influence on behaviour change are identified and brought together in a model that is intended 'as a memory aide for considering the different aspects of a complex situation and their many interactions' [23].

Reflecting on this model in the context of the data generated in this study, three areas for understanding the immediate context of RDT introduction were identified: characteristics of the innovation itself; involvement of purveyors; system antecedents for innovation; and system readiness for innovation. The additional areas identified in Greenhalgh's model of diffusion, adoption, implementation and consequences are, it can be argued, captured by the concepts of participation and reification of the innovation.

### The innovation

The innovation itself, RDTs for guiding anti-malarial prescription, appealed to health workers (all health workers were positive about the introduction of the tests and requested a continued supply) and had several characteristics that that enabled adoption of the test and had the potential to promote adherence to results. These were the simplicity of the RDTs, as stated by all participants, the trialability of the tests, as carried out by several participants in this study, and the observability of the accuracy of the RDTs. The latter attribute reflects health workers' ability to use clinical or microscopy based diagnoses of malaria as a reference point to judge the accuracy of the RDTs. However, this observability of the accuracy of the tests did not always lead to adherence with results given the poor sensitivity and specificity of both of these alternative diagnostic methods. Health workers identified many further relative advantages of the RDTs over other diagnostic methods, relating to the technical side of the test, its usefulness in diagnosis and its contribution to the health worker-patient relationship (Table 1).

**Table 1:** Relative advantages of RDTs from health worker perspective

Logistical advantages	Diagnostic advantages	Benefit to health worker-patient relationship
Nora, HFII 'From the beginning I like the RDT because **it works quicker**. By 15 minutes time you get a result.' Happy, HFIII 'It's **so fast **to know the patient is having malaria.' Flora, HFI 'Oh, **it's simple**, it doesn't waste time... The results come very simple and fast.' Anna, HFII 'When the result is out you go ahead and give the malaria treatment straight forward. But when the person goes to **lab he won't come back today **unless the following day or in three days time.' Fatima, HFIII 'Even at a place that there is **no electricity you can use **it to find out.' Jessica, HFIII 'I **do not need a microscope **that could be faulty to know if it is malaria or not.' Happy, HFIII 'It is **not like the microscopy **where you will say the person looking didn't see well or there might be some faults somewhere.' Esther, District Stakeholder 'It helps the lab men because it **takes a lot of the load off **them.'	Anna, HFII 'In fact, when I came first with the RDT I was very happy because **it can easily detect the malarial cases **... With the clinical [diagnosis] sometimes it is very difficult to diagnose ... so what we do is we still put the person on this routine drugs ... We the health workers will rather do the RDT and see whether the patient has malaria or not.' Jo, HFIII 'Ok the signs and symptoms are fine but ... I don't know why he is having the chill and the fever so we will ask him to go and in 30 minutes time, he'll come back with the RDT result. You get it? And **it will help you to diagnose**.' Jessica, HFIII 'It **helped me to think more**, because one thing I observed was that once the RDT tells me it is negative and then I think through almost all the time I get another differential diagnosis.'	Esther, District Stakeholder 'You get your diagnosis done; **doctor is happy patient is happy**.' Grace, HFII 'I started enjoying it because I realized that the patients it was **putting them at ease**. They like it they really like it.' Jessica, HFIII '**Everybody wants to know what is going on in the blood**. So when you say your sample is going to be taken then they are happy, they are satisfied.' Flora, HFI 'When you ask them to come to the test so that they will get the result and get treatment immediately **so that the person gets well **they are willing to do that.' Frances, HFI 'They like it in the first place that they don't go in for to the laboratory and **it's time saving**.' Anna, HFII 'The RDT facility that we are having here is making them to think that **they have a new thing here **... So some of them will say, "when I went to [HFII] Health Centre I went to lab". They have been calling the RDT "lab." "So they tested my blood and they said it is so so and so"' So they are happy with it.' Frida, HFI 'Most of them prefer having the results so that you **give the right treatment**.'

In addition to these inherent characteristics of RDTs that appealed to health workers, we identified a number of characteristics that were lacking or problematic in the innovation of RDTs in the context of this trial without supporting interventions. A number of relative risks of using RDTs were identified by participants. Underlying many of these risks was uncertainty over the accuracy of RDT results, derived from experience of patients with negative RDT results having malaria symptoms or positive lab results as well as reasons described in Figure 3.

Nora, HFII: *It's malaria there but is not showing. That's why everyday is negative negative negative... making mistake*.

--

Anna, HFII: *Other times too though they've said the person has a negative but the person still have the malaria symptoms and sometimes we want to argue about it and we allow the person to go and do lab somewhere. The person comes in with positive result. That's why I'm saying that it is not all that accurate*.

Risks related to potential inaccuracy of results revolved around the consequences of missing malaria, a disease seen as progressing to severe symptoms and death rapidly, a situation avoided by anti-malarial treatment, particularly if there was a chance the patient might not return with worsening symptoms,

Fatima, HFIII: '*Yes, we usually find it difficult especially where patient condition is alarming and you knowing that this could be malaria meanwhile this is proving negative'*.

---

Grace, HFII: '*You will never know whether he will return or not. Those who are far, far away they have their boundaries... So at times you have to use your discretion and you assess the person, 'no, no, I know you won't come back'. So for the benefit of the doubt you treat'*.

Risks of using RDTs extended to the potential damage to the health worker-patient relationship if symptoms worsened, or if the patient was dissatisfied with the diagnosis, particularly for RDT negative patients, as described in Figure 2. The time taken for testing with RDTs was also cited as problematic. In contrast to the advantage described above of being faster than microscopy, health workers also considered RDTs to be slow when comparing the tests with their routine of clinical diagnosis, and this was seen as a deterrent for patients to be tested,

Fatima, HFIII: *'The first problem I will say is it [the RDT] takes a lot of time for the result to be out... Some [patients] when they're late they will say 'please it will delay me'... They usually come back to us to complain, they will come back to complain to us that the test is delaying so if possible we should give them some treatment to go and come the following day for the result.'*

Whilst participants reported that the RDTs made treatment much easier for patients with positive results, a major problem cited by health workers using the RDTs was the diagnosis of RDT negative patients. Uncertainty over alternative diagnoses and treatments frequently led to anti-malarial treatment,

Grace, HFII: *'With the adults we've treated this we've treated that but still the client is not recovering and the test too says it's negative. So you're a bit confused so I treat. That's why I said earlier on that once a while even though it's negative but so often*

---

*Anna, HFII: With the negative ones, but if the client is still having the symptoms without any underlining cause, having the malaria symptoms, I think I will continue and treat the malaria'*.

Participants suggested that future roll out of RDTs should include further training to assist in the diagnosis and treatment of RDT negative treatments,

Jessica, HFIII: *'I think if we are emphasizing more on the differential diagnosis training, chronic care training, then they know what else to do when RDT is negative. And if we do that and then they are getting it very well, if they go back onto the field and they experience it, 'RDT was negative, I thought again, I got a differential diagnosis, the client didn't come back'. With experience they will get to know it's true after all. They are complaining; it's just because they don't know what else to do. They get frustrated when it's negative.'*

Treatment decisions were also influenced by existing interpretation of guidelines: in the absence messages to follow RDT results, the introduction of RDTs was compatible with and absorbed into existing practice. Because the RDTs were conducted by research staff in a separate room, health workers did not conduct the tests themselves, leading both to compatibility with existing microscopy testing systems but also lack of 'reinventability' of the RDTs into a local system for testing which may have generated more ownership of the tests and adaptation to the local context.

### Involvement of purveyors

The trial set out to assess the impact of the introduction of RDTs on practice in a setting with limited purveyor support, advocacy or supervision of RDTs in diagnostic practice. The deliberate lack of recommendations for treatment decisions was noted by health workers involved in the trial, who asked for advice from the research team, including for recommendations for RDT negative patients, and received a standard response to follow their own clinical judgement. Trust in the research team was apparent, and participants attempted to draw out recommendations from statements made by the team, sometimes leading to misinterpretation (Figure 1). The presence of members of the research team conducting the RDTs at the health centres was appreciated by some of the health workers interviewed, particularly for support in checking the accuracy of test results (Figure 1). This indicated that, in the absence of the research team, troubleshooting or supervision for both clinical and operational aspects of using the RDTs would be required.

At the end of the trial, a feedback meeting was held for the health workers involved in the trial. The results of the trial were not available but baseline results were presented, showing the extent of overdiagnosis by study participants. This meeting appeared to have a significant effect on all participants who had attended, particularly in encouraging them to become aware of their weaknesses in prescribing:

Flora, HFI: *'At the review the results that whatever we did here came out... The moment the results came then we were even ashamed (laughs) we were ashamed we were doing that, because we saw that we were doing the wrong thing rather instead of depending on the results of the RDT'*.

### System antecedents for innovation

This study of the introduction of RDTs included an assessment of the absorptive capacity for innovation and the receptiveness of the local context for change. The absorptive capacity for RDTs was affected by existing knowledge and mechanisms for learning. Baseline knowledge of malaria diagnosis was extensive given the long history and central place of the malaria in Ghanaian, and African, medical practice. The discourses highlighted as reifications reflecting non-significance of RDTs in case management in Figure 3 reflect existing mantras and cautions for malaria diagnosis with microscopy and a background preference for presumptive treatment. In addition, received knowledge of malaria was cited by all participants, describing the disease as highly prevalent, serious and progressing rapidly to severe illness and death. Experience with patients' expectations for malaria diagnosis and treatment was also long standing. This background knowledge and experience carried a lot of weight, leading to hesitation to adopt new practices with new tests. Formal mechanisms for in-service learning exist through continuing medical education programmes at the district level which may have indirectly enhanced capacity for absorbing innovations through bringing together health workers. However, formal mechanisms for learning at the local health facility level were few, limiting the capacity of directly facilitated change along with the RDT innovation.

The receptiveness of the local context for change to adopt RDTs appeared to be problematic. There was a discourse of being too busy to adopt new practices amongst many of the health workers interviewed,

Patricia, District Stakeholder: *'It's the system that makes you work that way. Who wouldn't want a laboratory confirmation?... but most of the time the facilities are just not there. then I will say that the work load too is another thing. The patients are many many and the lab is also overwhelmed with the numbers, ok, so sometimes we treat before the lab laboratory results are in'*.

Local leadership, important for local receptiveness for change, was also constrained. Whilst health workers did report trusting their superiors for advice, the medical officer in-charge of the largest health centre reported that previous quality improvement programmes had failed due to a weak management and promotion structure. Underlying the problems was the lack of power locally to incentivize staff to improve performance:

Esther, District Stakeholder: '*I think in a way managers have a limit on how to control staff behaviour because I don't pay them; somebody pays them, I manage, the powers are separated. It doesn't matter what you do at the clinic it doesn't affect your promotion. Because the promotions are done at the regional level in Accra.'*

### Local readiness for innovation

The local settings were ill-prepared for the changes required to adopt and adhere to RDTs. There was little tension for change, little time dedicated to adapting the local system for RDTs, a poor innovation-system fit in terms of treatment guidelines and a lack of capacity to evaluate tests.

When asked to reflect on the initial introduction of the RDTs, most health workers reported having been unaware that they were overdiagnosing malaria - reflecting a lack of tension for change to test-driven treatment. This was in part related to ambiguity over the role of diagnostic tests in malaria treatment guidelines. The conflict between the use of RDTs to guide diagnosis and national and international guidelines, such as for Integrated Management of Child Illnesses (IMCI) was problematic throughout the period of using the tests:

Jessica, HFIII: '*You know IMCI's kind of principle says if you have fever you treat for malaria for every under 5, that is the principle, that is the rule so that's a bit... yes contradictory, you don't know how to go in between that.'*

A key problem identified by health workers was the lack of ability to trust RDTs, citing that 'in a lot, at least one will fail' and 'nothing is 100%'. This doubt reflected a lack of capacity to evaluate the tests. There was feedback on quality control of the RDTs to health workers and their only method for assessing the quality of the tests was to follow up patients after treatment:

Grace, HFII: '*The problem is they don't usually come for follow up. You treat them and they go, 'come tomorrow, come in two days time,' and they never come. So it makes it very difficult because you have it in mind that you've seen this one and you are not very sure of what you've done. So come back and let me assess you and they don't come. So it makes it a bit difficult.'*

### RDT implementation framework

**Figure 4:**
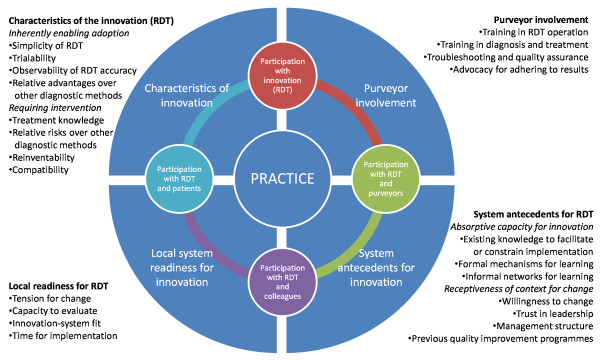
**Model of RDT implementation**. Adapted for RDT implementation from Wenger's (1998) and Greenhalgh *et al*'s (2004) models.

A visual representation of how RDTs were incorporated into practice in this study has been created together with the elements that affected this implementation, whether positively or negatively. Building on both Greenhalgh *et al*'s [23] and Wenger's [22] models, Figure 4 depicts the dynamic interplay between the innovation, systems and practice in the diffusion of the RDT innovation the practice of communities of health workers.

## Discussion

RDTs are now being deployed with the aim of improving the targeting of valuable anti-malarial drugs in rural areas of Africa. There is a push for policy to recommend the introduction of RDTs at both public and, increasingly, private sector health providers. There is however little evidence of the most effective methods for introducing RDTs. Technically RDTs work; operationally they often fall well short of their theoretical potential. In spite of calls for qualitative studies alongside quantitative evaluations of complex interventions [25], few published evaluations of how RDTs are understood and used by health workers exist. This qualitative study of the introduction of RDTs at rural health centres in Ghana has shown how health workers learnt and created meaning around the innovation of RDTs in the absence of deliberate supplementary behaviour change activities. Gaps in the delivery of the intervention that contributed to poor adherence with test results were identified together with priorities for future interventions to introduce RDTs into wide-scale clinical practice.

In contrast to models of behaviour change that perceive individuals as rational actors moving through a series of stages [24,26,27], this study found that learning was more dynamic: it occurred through interaction with others, including colleagues, researchers and patients, as well as individual experience with the rapid diagnostic tests. This finding is in line with research from Tanzanian hospitals where malaria was perceived to be easier and more acceptable to diagnose and treat than other diseases and that missing a malaria diagnosis was hard for health workers to justify, such that presumptive diagnosis was preferred. Four spheres of influence were identified as underlying these findings: peer pressure, patient preferences, diagnostic support and disease promotion. Peer and patient pressure were found to influence clinicians in their overdiagnosis of malaria in the face of microscopy results [28].

The importance of interactions with colleagues and with opinion leaders in the adoption of innovations, particularly with evidence based medicine, has been highlighted elsewhere [29-31]. However, interventions to change practice rarely harness this, most commonly preferring a didactic training model. In divergence with this, with the recognition of the central importance of informal networks in learning, Wenger's communities of practice (CoP) theory [22] has expanded from an analytical tool to an intervention tool. CoPs are now used as managerial techniques for improving organizational competitiveness in commercial settings and, to a lesser degree, as a medium for continuing professional development in health care settings [32,33]. In a recent systematic review, Li *et al *[34] report a variety of incarnations of CoPs in both business and health care with four common tenets for the groups: social interactions; knowledge-sharing; knowledge-creation and identity-building. Whilst CoPs have been reported to achieve these aims, the effect of CoPs on health outcomes has not been evaluated [34]. The application of CoPs to low-resource health care settings has not been reported but holds promise as an intervention to harness the influence of peers in defining practice.

The importance of interactions with patients in health worker learning and negotiation of practice has received less attention. The literature analysing health provider-patient relationships has classically critiqued the power relationship between provider and patient with the resulting restrictions for patients in terms of what they can say and how they can act [35-37] and this perspective remains informative when considering patient experiences [38,39]. The influence of patients on the development of provider practices has received less attention, reflecting long-standing assumptions of a close relationship between knowledge (as learnt in professional education) and practice. Some research has challenged this, pointing to the influence of patients on provider practice [40-42], including in overdiagnosing malaria when providers perceive patient demand [43]. However, the role of patients in the development of practice, particularly around new technologies, often remains implicit. This study found that health workers developed strategies to negotiate with patients over malaria test results: some leading to overuse of anti-malarials. The need to provide treatment, and explanations, to patients is clear in the provider-patient relationship. Negotiating a solution within a low-resource (in terms of tests and knowledge) setting is challenging. Interventions that have been successful in addressing these challenges include Balint groups. These groups have been used with general practitioners in various developed countries [44] to help deal with stressful work lives of GPs through reflection in peer groups. Balint groups focus on the physician-patient relationship, aiming to improve physicians' skills in handling their patients through reflection of their personal involvement and feelings during patient encounters [45]. Successes have been reported in improving attitudes towards work and towards patients and in reducing burn-out through participation in these groups [46]. Interventions based on these same tenets have been piloted in a number of African settings with considerable impact on job motivation (Ane Haaland, unpublished work). Such groups could prove important in the significant shift from anti-malarial overuse to treatment or non-treatment of alternative causes of symptoms.

In this study, we found that many of the characteristics of the innovation and implementing context were not conducive to the adoption of RDTs. Following the categories set out by Greenhalgh *et al *[23], some characteristics of RDTs were identified as inherently conducive to behaviour change: lack of complexity of the test; relative advantages compared to alternative diagnostics; compatibility with previous practice in diagnostics; observability of results and consequences; and trialability. However, low uptake of RDT results may reflect the lack of additional interventions in supporting knowledge required, particularly for diagnosing alternative causes of symptoms; reinvention of logistics of RDT use locally; addressing risks to relationships with patients; and augmentation in terms of trouble shooting for RDT quality and case management. Thus far, much investment has gone into ensuring safety and accuracy of RDT use, with the development of effective pictorial job aids for using RDTs [47] and transport, storage, stock management and waste management of the tests [48]. A comparable level of investment into detail and harmonization needs to be given to the adoption of RDTs to clinical practice.

In terms of the implementing context, this study found variation between health workers and health facilities in the absorptive capacity for innovation and receptiveness of context for change. Existing conceptualizations of malaria risk and diagnosis formed a barrier to the use of RDT results and the lack of formal mechanisms for learning, weak management structure and experience of previous failed improvement programmes are likely to have been insurmountable for many participants. Supportive interventions need to address existing conceptualizations through more formalized channels of learning, building on the informal networks apparent in this study. Such methods may reduce the necessity for strong management and top-down incentives, factors that will need to be addressed at a wider health systems level. The support for Communities of Practice groups may be an avenue for situated learning and adaptation of the RDT innovation existing paradigms of practice.

This study found that local systems were unprepared for supporting a new innovation. The surprise of many health workers that they were overdiagnosing malaria showed a relative absence of 'tension for change'. In addition, little time was dedicated to implementing the innovation, there was no local capacity to evaluate the innovation, there was little support or advocacy from purveyors and there was confusion over guidelines, leading to a poor innovation-system fit. If tension for change were created, by media coverage, involvement in prevalence studies or simply through use of the RDTs, this study's results suggest that these additional supportive interventions would still need to be addressed. Those in charge of health facilities should be encouraged to set aside time and space for CoPs in their health facilities to address challenges of RDT implementation through setting up their own mechanisms of evaluation and clinical troubleshooting. In addition, this study reiterates the need for clearer guidelines for malaria diagnosis and treatment from policy makers [49].

There are a number of limitations in this study. Firstly, this qualitative research was conducted one month after the end of the intervention trial, with the aim to understand health worker perspectives without influencing their practice in the trial, but with the consequence that participants had already taken part in a feedback session about the trial during which their overdiagnosis of malaria was discussed. Although this feedback session did not give the results from the trial, it did provide an occasion for participants to meet and reflect on their experiences. This is likely to have affected the results discussed here, as described in Figure 1, and may have potentially led to increased uptake and adherence to RDT results had the tests continued to be available. Secondly, the trial randomized individual patients to microscopy or RDT, or to RDT or clinical diagnosis. This meant that the health workers involved were unable to choose the method of testing and were asked to make diagnostic decisions on the basis of either of two methods. Together with the knowledge that the RDTs were temporarily available for the trial period, these factors may have affected how participants became accustomed to the new tests, including a lack of structural support for wholesale changes to RDTs. Finally, the generalizability of this study's results may be questioned. Thirteen in-depth interviews were conducted with a purposive sample of prescribers and stakeholders in one district in Ghana. The sample size was sufficient to capture recurring ideas amongst participants, however, and whilst reified phrases may have been locally specific, the concepts have been related to wider literature and theory and the authors believe these reflect concepts that should be considered in any introduction of innovations such as rapid diagnostic tests for malaria.

To support the introduction of RDTs, three sets of recommendations can be made for purveyors. Firstly, RDT scale-up must be accompanied by unambiguous and consistent guidelines, clear messages to providers and well-designed training to combat the long-standing paradigm of presumptive treatment. This training must specifically include skills in diagnosing alternative causes of malaria-like symptoms and dealing with malaria-negative patients. Secondly, RDTs can be augmented through advocacy for RDTs to patients and providers, through trouble-shooting schemes with RDT or diagnostic experts, and through the establishment of timely feedback of RDT quality control results to providers. Thirdly, local health facilities must be prepared and supported for the introduction of RDTs. Preparation can occur through raising awareness of the need for change, identifying leaders and working with health workers to identify logistical changes needed for adoption of RDTs. The results of this study suggest on-going support may be effective through the establishment of communities of practice to discuss changes and implications for practice, and/or Balint-style facilitated groups to encourage health workers to reflect on their interactions with patients to enable them to become confident prescribers of good quality health care, reflected in satisfied and healthy patients.

## Conclusion

This qualitative study added an in-depth evaluation component to a randomized controlled trial of rapid diagnostic tests for malaria compared with microscopy or clinical diagnosis. The study evaluated the uptake of this technological innovation in the absence of deliberate support for its adaptation into prescribing practice with the goal to learn lessons for future implementation of RDTs. Health workers were found to have learned and practiced through participation and reification: interaction with the test itself, with the RDT and peers, with the RDT and patients and with the research team. A number of contextual factors were identified to have aided and constrained the adherence of health workers to negative RDT results. If RDTs results are to be adhered to, more attention needs to be given to supporting interventions to help providers to change a long-standing paradigm of malaria overdiagnosis.

## Competing interests

The authors declare that they have no competing interests.

## Authors' contributions

CC designed the evaluation, carried out the data collection and analysis and drafted the manuscript. CW contributed to evaluation design and drafting the manuscript. EA contributed to evaluation design, data collection, interpretation and drafting of the manuscript. All authors read an approved the final manuscript.

## References

[B1] Hopkins H Asiimwe C Bell D Access to antimalarial therapy: accurate diagnosis is essential to achieving long term goals BMJ 2009 339 b2606 10.1136/bmj.b2606 19584113

[B2] Whitty C Chandler C Ansah E Leslie T Staedke S Deployment of ACT antimalarials for treatment of malaria: challenges and opportunities Malaria Journal 2008 7 S7 10.1186/1475-2875-7-S1-S7 19091041 PMC2604871

[B3] Reyburn H Mbatia R Drakeley C Carneiro I Mwakasungula E Mwerinde O Saganda K Shao J Kitua A Olomi R Overdiagnosis of malaria in patients with severe febrile illness in Tanzania: a prospective study BMJ 2004 329 1212 10.1136/bmj.38251.658229.55 15542534 PMC529364

[B4] Amexo M Tolhurst R Barnish G Bates I Malaria misdiagnosis: effects on the poor and vulnerable Lancet 2004 364 1896 1898 10.1016/S0140-6736(04)17446-1 15555670

[B5] Hume JC Barnish G Mangal T Armazio L Streat E Bates I Household cost of malaria overdiagnosis in rural Mozambique Malar J 2008 7 33 10.1186/1475-2875-7-33 18282270 PMC2279141

[B6] Font F Alonso Gonzalez M Nathan R Kimario J Lwilla F Ascaso C Tanner M Menendez C Alonso PL Diagnostic accuracy and case management of clinical malaria in the primary health services of a rural area in south-eastern Tanzania Trop Med Int Health 2001 6 423 428 10.1046/j.1365-3156.2001.00727.x 11422955

[B7] Krause G Sauerborn R Comprehensive community effectiveness of health care. A study of malaria treatment in children and adults in rural Burkina Faso Ann Trop Paediatr 2000 20 273 282 11219164 10.1080/02724936.2000.11748147

[B8] Van Dillen J De Jager AJ De Jong I Wendte JF Overdiagnosis of malaria in hospitalized patients in Namibia Trop Doct 2007 37 185 186 10.1258/004947507781524845 17716517

[B9] Makani J Matuja W Liyombo E Snow RW Marsh K Warrell DA Admission diagnosis of cerebral malaria in adults in an endemic area of Tanzania: implications and clinical description QJM 2003 96 355 362 10.1093/qjmed/hcg059 12702784 PMC5612393

[B10] Lubell Y Reyburn H Mbakilwa H Mwangi R Chonya S Whitty CJM Mills A The impact of response to the results of diagnostic tests for malaria: cost-benefit analysis Bmj 2008 336 202 205 10.1136/bmj.39395.696065.47 18199700 PMC2213875

[B11] Msellem MI Martensson A Rotllant G Bhattarai A Stromberg J Kahigwa E Garcia M Petzold M Olumese P Ali A Bjorkman A Influence of rapid malaria diagnostic tests on treatment and health outcome in fever patients, Zanzibar: a crossover validation study PLoS Med 2009 6 e1000070 10.1371/journal.pmed.1000070 19399156 PMC2667629

[B12] Williams HA Causer L Metta E Malila A O'Reilly T Abdulla S Kachur SP Bloland PB Dispensary level pilot implementation of rapid diagnostic tests: an evaluation of RDT acceptance and usage by providers and patients--Tanzania, 2005 Malar J 2008 7 239 10.1186/1475-2875-7-239 19019233 PMC2613413

[B13] D'Acremont V Massive reduction of antimalarial prescription after Rapid Diagnostic Test implementation in Dar es Salaam, Tanzania Presented at MIM Pan-African Malaria Conference. Nairobi, Kenya 2009

[B14] Counihan H Malaria rapid diagnosis performance by community health workers, Zambia Presented at MIM Pan-African Malaria Conference. Nairobi, Kenya 2009

[B15] Hopkins H Effectiveness and safety of training in fever case management and RDT use at health centres in Uganda Presented at American Society of Tropical Medicine & Hygiene 57th Annual Meeting. New Orleans 2008

[B16] Ansah EK Narh-Bana S Epokor M Akanpigbiam S Quartey AA Gyapong J Whitty CJ Rapid testing for malaria in settings where microscopy is available and peripheral clinics where only presumptive treatment is available: a randomised controlled trial in Ghana BMJ 2010 340 c930 10.1136/bmj.c930 20207689 PMC2833239

[B17] Reyburn H Mbakilwa H Mwangi R Mwerinde O Olomi R Drakeley C Whitty CJM Rapid diagnostic tests compared with malaria microscopy for guiding outpatient treatment of febrile illness in Tanzania: randomised trial Bmj 2007 334 403 10.1136/bmj.39073.496829.AE 17259188 PMC1804187

[B18] Skarbinski J Ouma PO Causer LM Kariuki SK Barnwell JW Alaii JA de Oliveira AM Zurovac D Larson BA Snow RW Effect of malaria rapid diagnostic tests on the management of uncomplicated malaria with artemether-lumefantrine in Kenya: a cluster randomized trial Am J Trop Med Hyg 2009 80 919 926 19478249

[B19] Hamer DH Ndhlovu M Zurovac D Fox M Yeboah-Antwi K Chanda P Sipilinyambe N Simon JL Snow RW Improved diagnostic testing and malaria treatment practices in Zambia JAMA 2007 297 2227 2231 10.1001/jama.297.20.2227 17519412 PMC2674546

[B20] Bisoffi Z Sirima BS Angheben A Lodesani C Gobbi F Tinto H Ende J Van den Rapid malaria diagnostic tests vs. clinical management of malaria in rural Burkina Faso: safety and effect on clinical decisions. A randomized trial Trop Med Int Health 2009 14 491 498 10.1111/j.1365-3156.2009.02246.x 19222821

[B21] Strauss A Corbin J Basics of qualitative research: Grounded theory procedures and techniques 1990 London: Sage Publications

[B22] Wenger E Communities of Practice. Learning, meaning, and identity 1998 New York: Cambridge University Press

[B23] Greenhalgh T Robert G Macfarlane F Bate P Kyriakidou O Diffusion of innovations in service organizations: systematic review and recommendations Milbank Q 2004 82 581 629 10.1111/j.0887-378X.2004.00325.x 15595944 PMC2690184

[B24] Rogers EM Diffusion of Innovations 1995 4 New York: The Free Press

[B25] Lewin S Glenton C Oxman AD Use of qualitative methods alongside randomised controlled trials of complex healthcare interventions: methodological study BMJ 2009 339 b3496 10.1136/bmj.b3496 19744976 PMC2741564

[B26] Prochaska JO DiClemente CC Hersen M, Eisler RM, Miller PM Stages of change in the modification of problem behaviours Progress in Behaviour Modification 1992 Sycamore: Sycamore Publishing Co

[B27] Slotnick HB How doctors learn: physicians' self-directed learning episodes Acad Med 1999 74 1106 1117 10.1097/00001888-199910000-00014 10536633

[B28] Chandler CI Jones C Boniface G Juma K Reyburn H Whitty CJ Guidelines and mindlines: why do clinical staff over-diagnose malaria in Tanzania? A qualitative study Malar J 2008 7 53 10.1186/1475-2875-7-53 18384669 PMC2323020

[B29] Mittman BS Tonesk X Jacobson PD Implementing clinical practice guidelines: social influence strategies and practitioner behavior change QRB Qual Rev Bull 1992 18 413 422 1287523 10.1016/s0097-5990(16)30567-x

[B30] Greer AL The state of the art versus the state of the science. The diffusion of new medical technologies into practice Int J Technol Assess Health Care 1988 4 5 26 10.1017/S0266462300003202 10287114

[B31] Grimshaw JM Eccles MP Greener J Maclennan G Ibbotson T Kahan JP Sullivan F Is the involvement of opinion leaders in the implementation of research findings a feasible strategy? Implement Sci 2006 1 3 10.1186/1748-5908-1-3 16722572 PMC1436013

[B32] Li LC Grimshaw JM Nielsen C Judd M Coyte PC Graham ID Evolution of Wenger's concept of community of practice Implement Sci 2009 4 11 10.1186/1748-5908-4-11 19250556 PMC2654669

[B33] Parboosingh JT Physician communities of practice: where learning and practice are inseparable J Contin Educ Health Prof 2002 22 230 236 10.1002/chp.1340220407 12613058

[B34] Li LC Grimshaw JM Nielsen C Judd M Coyte PC Graham ID Use of communities of practice in business and health care sectors: A systematic review Implement Sci 2009 4 27 10.1186/1748-5908-4-27 19445723 PMC2694761

[B35] Stimson GV Webb B Going to See the Doctor: the Consultation Process in General Practice 1975 London: Routledge & Kegan Paul

[B36] Johnson T Professions and Power 1972 Hampshire: Macmillan Education Ltd

[B37] Foucault M Discipline and Punish: The Birth of the Prison 1977 Harmondsworth: Penguin

[B38] Mogensen HO Finding a path through the health unit: practical experience of Ugandan patients Med Anthropol 2005 24 209 236 10.1080/01459740500182659 16081334

[B39] Bury M Researching patient-professional interactions J Health Serv Res Policy 2004 9 Suppl 1 48 54 10.1258/135581904322724130 15006228

[B40] Howteerakul N Higginbotham N Freeman S Dibley MJ ORS is never enough: physician rationales for altering standard treatment guidelines when managing childhood diarrhoea in Thailand Soc Sci Med 2003 57 1031 1044 10.1016/S0277-9536(02)00478-1 12878103

[B41] Mangione-Smith R McGlynn EA Elliott MN Krogstad P Brook RH The relationship between perceived parental expectations and pediatrician antimicrobial prescribing behavior Pediatrics 1999 103 711 718 10.1542/peds.103.4.711 10103291

[B42] Greer AL Goodwin JS Freeman JL Wu ZH Bringing the patient back in. Guidelines, practice variations, and the social context of medical practice Int J Technol Assess Health Care 2002 18 747 761 10.1017/S0266462302000569 12602076

[B43] Chandler CI Mwangi R Mbakilwa H Olomi R Whitty CJ Reyburn H Malaria overdiagnosis: is patient pressure the problem? Health Policy Plan 2008 23 170 178 10.1093/heapol/czm046 18321889

[B44] Salinsky J The Balint movement worldwide: present state and future outlook: a brief history of Balint around the world Am J Psychoanal 2002 62 327 335 10.1023/A:1021188731808 12512676

[B45] Balint M The Doctor, his Patient and the Illness 1963 2 London: Churchill Livingstone

[B46] Kjeldmand D Holmstrom I Balint groups as a means to increase job satisfaction and prevent burnout among general practitioners Ann Fam Med 2008 6 138 145 10.1370/afm.813 18332406 PMC2267420

[B47] Harvey SA Jennings L Chinyama M Masaninga F Mulholland K Bell DR Improving community health worker use of malaria rapid diagnostic tests in Zambia: package instructions, job aid and job aid-plus-training Malar J 2008 7 160 10.1186/1475-2875-7-160 18718028 PMC2547110

[B48] WHO-WPRO, USAID|DELIVER PROJECT, Foundation for Innovative New Diagnostics, Roll Back Malaria Partnership, President's Malaria Initiative, UNICEF Transporting, Storing, and Handling Malaria Rapid Diagnostic Tests in Health Clinics 2009 Arlington, Va: USAID | DELIVER PROJECT, Task Order 3; and Manila: WHO-WPRO

[B49] D'Acremont V Lengeler C Genton B Stop ambiguous messages on malaria diagnosis BMJ 2007 334 489 10.1136/bmj.39143.024838.1F 17347193 PMC1819502

